# An Efficient CRT-Base Power-of-Two Scaling in Minimally Redundant Residue Number System

**DOI:** 10.3390/e24121824

**Published:** 2022-12-14

**Authors:** Mikhail Selianinau, Yuriy Povstenko

**Affiliations:** Department of Mathematics and Computer Sciences, Faculty of Science and Technology, Jan Dlugosz University in Czestochowa, al. Armii Krajowej 13/15, 42-200 Czestochowa, Poland

**Keywords:** residue number system, Chinese Remainder Theorem, modular arithmetic, rank of a number, power-of-two scaling

## Abstract

In this paper, we consider one of the key problems in modular arithmetic. It is known that scaling in the residue number system (RNS) is a rather complicated non-modular procedure, which requires expensive and complex operations at each iteration. Hence, it is time consuming and needs too much hardware for implementation. We propose a novel approach to power-of-two scaling based on the Chinese Remainder Theorem (CRT) and rank form of the number representation in RNS. By using minimal redundancy of residue code, we optimize and speed up the rank calculation and parity determination of divisible integers in each iteration. The proposed enhancements make the power-of-two scaling simpler and faster than the currently known methods. After calculating the rank of the initial number, each iteration of modular scaling by two is performed in one modular clock cycle. The computational complexity of the proposed method of scaling by a constant $S_{l}=2^{l}$ associated with both required modular addition operations and lookup tables is estimeted as *k* and 2k+1, respectively, where *k* equals the number of primary non-redundant RNS moduli. The time complexity is $\left\lceil \log_{2}k\right\rceil +l$ modular clock cycles.

## 1. Introduction

Nowadays, high-performance computing is progressing extremely rapidly. This makes qualitatively new demands to designed number-theoretic methods and computational algorithms. That is why creating fundamentally new and efficient computing tools for fast and reliable parallel data processing is especially important. Modular computational structures occupy a special place among them. Modular arithmetic, i.e., the arithmetic of RNS, creates their mathematical basis.

The inherent parallelism and carry-free properties of RNS provide a high potential for accelerating arithmetic operation compared with conventional weighted number systems (WNS). The main advantage of RNS consists of its unique ability to decompose large integer numbers into a set of small residues and to process them in parallel in independent modular channels.

A steadily growing interest in RNS arithmetic as a unique means of carrying out high-speed calculations stimulates developments focused on providing a fundamentally new performance level when carrying out large volumes of time-consuming calculations. The modular arithmetic has attracted the considerable attention of researchers and developers in number-theoretic methods [1,2,3], computer technology [4,5], digital signal and image processing [3,5,6,7,8], cryptography [5,8,9,10], computer networks and communication systems [3,5], and other areas [9].

In this regard, one of the most promising ways in the specified area is the development of high-speed parallel modular computational structures as well as the enhancement of their functionality and optimization. In this case, the main optimization criteria are the minimum redundancy of data coding, the execution time minimization of implemented computational procedures, and the throughput maximization of the corresponding computational structures.

As is known, compared with WNS, the residue code of a number does not explicitly contain information about its integer value. Therefore, in RNS arithmetic, the implementation of operations, which require the estimating of the integer value of a number by its residue code, i.e., the evaluation of number position in the operating range, encounters specific difficulties. Such operations, in contrast to modular ones, are called non-modular.

The positional characteristics of the residue code such as core function, the rank of a number, interval index, and others, and the associated forms of number representation are of great importance for designing algorithms of non-modular operations [1,5,7,8]. The computational complexity of calculating the used positional characteristics ultimately determines the efficiency of the corresponding configuration of RNS arithmetic.

Division is one of the most complex arithmetic operations. Even in computers operating in a positional system, this operation stands apart, and its execution requires much more time than most elementary operations. In RNS arithmetic, the hardships with division operations are related to the non-modular character of this operation. This means that the residue of the quotient concerning primary RNS modulus is determined not only by the dividend and divisor residues, and it is necessary to get the additional information, in one form or another, about the integer values of the dividend and divisor [1,7]. It is no coincidence that many publications are devoted to the problem of modular division, for example [11,12,13,14,15,16,17,18,19].

Along with the general division, modular scaling, i.e., the division of the RNS number by a constant, is a commonly used operation [3,5,8,10]. This operation plays a fundamental role in constructing residue arithmetic algorithms and is of great practical importance. The need for scaling is due to several tasks, for example, to round the floating point numbers with the residue representation of the mantissa and to reduce the dynamic range in digital signal processing and long-word-length cryptography. In addition, scaling by a power of two is often one of the integral steps of more complex non-modular operations, for example, in the method of general modular division [19,20].

Thus, developing novel approaches and methods for fast scaling is highly important in high-performance computing based on parallel algorithmic structures of RNS, especially for high-speed implementing digital signal processing applications and public-key cryptosystems. That should make it possible to widely use modular arithmetic in various priority areas of science and technology.

In the paper, we present a new approach to the power-of-two scaling based on using minimal redundancy of residue code, the rank form of a number, and fast calculation of the rank characteristic at each iteration of the scaling procedure. Compared with the conventional non-redundant RNS, the proposed method makes it possible to optimize and speed up the non-modular scaling operation and concurrently reduces its computational complexity to a large extent.

The paper is structured as follows. Section 2 discusses the basic theoretical concepts of the research. Section 3 presents the known approaches to rank calculation. Section 4 and Section 5 describe the RNS scaling algorithms, and the mathematical basis of the rank calculation in the bisection scaling method. Section 6 presents a novel power-of-two scaling algorithm and a numerical example. Section 7 provides discussion and Section 8 concludes the paper.

## 2. The Basic Concepts of RNS Arithmetic

Abstract algebra and number theory [21,22] constitute the theoretical foundation of RNS arithmetic.Traditionally, the apparatus of congruences is used for the mathematical formalization of an RNS with integer ranges. At the same time, Euclid’s Division Lemma plays a fundamental role in building an RNS of the concerned type. For the ring **Z** of integers, it is formulated as follows.

**Lemma** **1 (Euclid’s Division Lemma).** *For any X∈Z and a positive integer m, there exists a unique pair of integers Q,R such that*

(1) X=Qm+R,

*where $R\in Z_{m}=\left\{0,1,\ldots ,m-1\right\}$.*

On the set **Z** of integers, a non-redundant RNS is defined using pairwise prime moduli $m_{1},m_{2},\ldots ,m_{k}$ $\left(k>1\right)$ by the mapping $Z\to Z_{m_{1}}\times Z_{m_{2}}\times \cdots \times Z_{m_{k}}$, which assigns to each X∈Z the *k*-tuple $\left(\chi_{1},\chi_{2},\ldots ,\chi_{k}\right)$ of least nonnegative residues $\chi_{i}={\left|X\right|}_{m_{i}}$ of dividing *X* by $m_{i}$$\left(i=1,2,\ldots ,k\right)$. At the same time, the notation $X=\left(\chi_{1},\chi_{2},\ldots ,\chi_{k}\right)$ is used.

The residue code $\left(\chi_{1},\chi_{2},\ldots ,\chi_{k}\right)$ corresponds to the set of all integers *X* satisfying the system of simultaneous linear congruences

(2) $$\left\{\begin{array}{c} X\equiv \chi_{1}\left(\mathrm{mod}m_{1}\right),\\ X\equiv \chi_{2}\left(\mathrm{mod}m_{2}\right),\\ \ldots \\ X\equiv \chi_{k}\left(\mathrm{mod}m_{k}\right) \end{array}\right.$$

The following statement is true [9,23,24].

**Theorem** **1 (Chinese Remainder Theorem).** *Let the moduli $m_{1},m_{2},\ldots ,m_{k}$ be pairwise prime, and let $M_{k}=\prod_{i=1}^{k}m_{i}$, $M_{i,k}=M_{k}/m_{i}$, $\mu_{i,k}={\left|M_{i,k}^{-1}\right|}_{m_{i}}$$\left(i=1,2,\ldots ,k\right)$. Then the system of congruences *(2)* has a unique solution, the class of residues modulo $M_{k}$, defined by the congruence*

(3) $$X\equiv \sum_{i=1}^{k}M_{i,k}\mu_{i,k}\chi_{i}\left(\mathrm{mod}M_{k}\right).$$

The practical application of the RNS assumes that each residue code $\left(\chi_{1},\chi_{2},\ldots ,\chi_{k}\right)$ must correspond only to one integer number. Therefore, certain sets of representatives of residue classes are used as the number range to ensure required single-valued correspondence. Since in the given RNS it is possible to represent $M_{k}$ integers, the set $Z_{M_{k}}=\left\{0,1,\ldots ,M_{k}-1\right\}$ is usually used in computer applications as an RNS operating range.

Because of the above, we define modular coding as a mapping $\Phi_{RNS}:Z_{M_{k}}\to Z_{1}\times Z_{2}\times \cdots \times Z_{m_{k}}$, which assigns a residue code $\left(\chi_{1},\chi_{2},\ldots ,\chi_{k}\right)$ to each $X\in Z_{M_{k}}$.

The decoding mapping $\Phi_{RNS}^{-1}:Z_{1}\times Z_{2}\times \cdots \times Z_{m_{k}}\to Z_{M_{k}}$ based on the CRT (3) executes according to the rule

(4) $$X={\left|\sum_{i=1}^{k}M_{i,k}\mu_{i,k}\chi_{i}\right|}_{M_{k}}.$$

Applying Euclid’s Division Lemma (1), we can write

(5) $$\mu_{i,k}\chi_{i}={\left|\mu_{i,k}\chi_{i}\right|}_{m_{i}}+\left\lfloor \frac{\mu_{i,k}\chi_{i}}{m_{i}}\right\rfloor m_{i}=\chi_{i,k}+\left\lfloor \frac{\mu_{i,k}\chi_{i}}{m_{i}}\right\rfloor m_{i},$$

where $\chi_{i,k}$ is a normalized residue modulo $m_{i}$:

(6) $$\chi_{i,k}={\left|\mu_{i,k}\chi_{i}\right|}_{m_{i}}\left(i=1,2,\ldots ,k\right),$$

$\left\lfloor x\right\rfloor$ denotes the largest integer less than or equal to *x*.

Substituting (5) into (4), and taking into consideration (6), we have

$$X={\left|\sum_{i=1}^{k}M_{i,k}\chi_{i,k}+M_{k}\sum_{i=1}^{k}\left\lfloor \frac{\mu_{i,k}\chi_{i}}{m_{i}}\right\rfloor \right|}_{M_{k}}$$

that is equivalent to

(7) $$X={\left|\sum_{i=1}^{k}M_{i,k}\chi_{i,k}\right|}_{M_{k}}.$$

Since the summands in (7) have narrower change bounds, the use of (4), which is a normalized analog of (1), is preferable for constructing RNS arithmetic.

Equation (7) is called the CRT-form of representing the integer $X=\left(\chi_{1},\chi_{2},\ldots ,\chi_{k}\right)$ from the RNS number range $Z_{M_{k}}$.

The mapping $\Phi_{RNS}$ is an isomorphism concerning the basic arithmetic operations. The operation $\circ \in \left\{+,-,\times \right\}$ on arbitrary elements *A* and *B* given by their residue codes $A=\left(\alpha_{1},\alpha_{2},\ldots ,\alpha_{k}\right)$ and $B=\left(\beta_{1},\beta_{2},\ldots ,\beta_{k}\right)$ is carried out by the rule

$$A\circ B=\left(\alpha_{1},\alpha_{2},\ldots ,\alpha_{k}\right)\circ \left(\beta_{1},\beta_{2},\ldots ,\beta_{k}\right)=$$

(8) $$=\left({\left|\alpha_{1}\circ \beta_{1}\right|}_{m_{1}},{\left|\alpha_{2}\circ \beta_{2}\right|}_{m_{2}},\ldots ,{\left|\alpha_{k}\circ \beta_{k}\right|}_{m_{k}}\right),$$

where $\alpha_{i}={\left|A\right|}_{m_{i}},\beta_{i}={\left|B\right|}_{m_{i}},i=1,2,\ldots ,k$.

In the RNS, according to (8), the modular addition, subtraction, and multiplication are performed independently for each modulus $m_{i}$$\left(i=1,2,\ldots ,k\right)$. It must be noted that (8) is correct only if the result A∘B of the arithmetic operation does not go beyond the RNS number range, i.e., if $A\circ B\in Z_{M_{k}}$.

The RNS inherent code parallelism illustrated by (8), which consists of the decomposition of arithmetic operations on integers *A* and *B* into independent small word length operations on the like digits $\alpha_{i}$ and $\beta_{i}$ of residue code, is the main advantage of modular arithmetic compared with the arithmetic of weighted number systems (WNS). Realizing this advantage to the fullest extent is a key strategic goal of all computer applications in the RNS.

As is known, in contrast to the positional code, the residue code $\left(\chi_{1},\chi_{2},\ldots ,\chi_{k}\right)$ of the number *X* does not explicitly contain information about its value. Therefore, the implementation in the RNS arithmetic operations that require calculating the so-called positional characteristics which give information about the numbers location in the RNS range $Z_{M_{k}}$ encounters specific difficulties. Such procedures, in contrast to modular ones, are called non-modular.

The efficiency factor of RNS arithmetic, to a decisive extent, is determined by the optimality of the applied non-modular procedures. At the same time, the main factor that has the most impact on the quality indicators of algorithms for non-modular operations is the computational complexity of calculating the positional characteristics of the residue code and related integer representation forms.

As for Equation (7), its direct application as the general form of integers for building non-modular procedures is practically impossible due to the complexity of straightforward implementation, especially in the case of large $M_{k}$. At the same time, the use of the specific positional characteristics enables us to obtain from (7) the relevant forms of integer representation, which have good implementation properties and make it possible to overcome the problem of time-consuming addition operations modulo $M_{k}$.

As follows from (7), the difference

$$\sum_{i=1}^{k}M_{i,k}\chi_{i,k}-X$$

is a multiple of $M_{k}$. Hence, the following equality holds

(9) $$X=\sum_{i=1}^{k}M_{i,k}\chi_{i,k}-\rho_{k}\left(X\right)M_{k}.$$

The positional characteristic $\rho_{k}\left(X\right)$ is called a rank of the number *X*. In essence, the rank $\rho_{k}\left(X\right)$ is a CRT reconstruction coefficient that indicates how many times the upper bound $M_{k}$ of the number range is exceeded when the integer value of the number *X* is calculated by its residue code $\left(\chi_{1},\chi_{2},\ldots ,\chi_{k}\right)$.

Equation (9) is called a rank form of the integer *X*.

From (9), it also follows that the rank $\rho_{k}\left(X\right)$ is a quotient of the integer division of $X_{k}$ by $M_{k}$.

Hence, we obtain

(10) $$\rho_{k}\left(X\right)=\left\lfloor \frac{1}{M_{k}}\sum_{i=1}^{k}M_{i,k}\chi_{i,k}\right\rfloor =\left\lfloor \sum_{i=1}^{k}\frac{\chi_{i,k}}{m_{i}}\right\rfloor .$$

Therefore, since $\chi_{i,k}\in Z_{m_{i}}$$\left(i=1,2,\ldots ,k\right)$, the inequality $0\leq \rho_{k}\left(X\right)\leq k-1$ holds.

Compared with (7), Equation (9) does not contain time-consuming reduction modulo $M_{k}$. Therefore, designing non-modular procedures in RNS arithmetic on the basis of the rank form has a substantial lead over the canonical CRT implementation.

## 3. The Approaches Currently Used to Calculate the Rank of a Number

First, the rank of a number as a main RNS integral characteristic has been studied in [1], and later in [2]. The rank evaluation algorithm consists of a slow *k*-step iterative procedure of sequential additions large modulo of specific constants defined by the chosen RNS moduli-set $\left\{m_{1},m_{2},\ldots ,m_{k}\right\}$. Moreover, the upper bound of the rank r(X) depends on the values of the weights $\mu_{1,k},\mu_{2,k},\ldots ,\mu_{k,k}$ (see (4)), and can be sufficiently large for most moduli-sets suitable for practical use. If we assume that the processing of such long *L*-bit word-length numbers $L=\left(\left\lceil \log_{2}M_{k}\right\rceil \right)$ is comparable in time with *k* operations on the small residues, then the complexity of this method is equal to $O\left(k^{2}\right)$. Because of that, the given approach to the rank calculation is time-consuming and practically unacceptable for high-performance computing due to its computational complexity, especially when using huge $M_{k}$.

The so-called “extra modulus method” for rank calculation has been proposed in [25]. It rearranges the canonical CRT implementation to an exact integer equation, i.e., the same form as (9). To be able to retrieve the value of the CRT reconstruction coefficient, i.e., the rank of a number, the extra-modulus $m_{e}$ must satisfy the following conditions: $m_{e}>k$, and $m_{e}$ is any integer prime to $M_{k}$. In this way, the slow and challenging addition modulo $M_{k}$ in the straightforward CRT implementation is replaced by subtraction and multiplication modulo $m_{e}$. Thus, we have an extra modular channel for rank calculation. This method works well and correctly when it assumes that proper redundant residue ${\left|X\right|}_{m_{e}}$ is available. Hence, the “extra modulus method” is suitable for the base extension operation. At the same time, when the number under consideration results from the modular addition or subtraction operation [26], it cannot be used owing to eventual overflow or underflow, respectively. Thus, in such a case, the exact value of ${\left|X\right|}_{m_{e}}$ is not available. Therefore, this method is not applicable for sign determination and magnitude comparison of two numbers in RNS.

A different approach to evaluating the CRT reconstruction coefficient is proposed in [27,28,29]. The main idea of the so-called ”fractional domain method” consists in the representation of the reconstruction coefficient *r* as an integer part of a sum of at most *k* proper fractions (see (10)). The value *r* is recursively estimated by approximating terms of a fraction $\chi_{i,k}/m_{i}$. To avoid division by the modulus $m_{i}$ in the fraction, the denominator $m_{i}$ is replaced by $2^{n}$$\left(m_{i}<2^{n}\right)$, while the numerator $\chi_{i,k}$ is approximated by its most significant υ bits $\left(\upsilon <n\right)$$\left(i=1,2,\ldots ,k\right)$. Since the division by powers of 2 is equivalent to simple shifts, then the calculation of *r* can be implemented by addition only.

The main drawbacks of this method consist of the following. First of all, full-precision fractional computations are required. In any case, such calculations are slower than operating on smaller word-length, and the full-precision fractional bits require substantial storage. On the other hand, the number of iterations required is of the order of the bit-length needed for the approximation. For example, the method employing a fractional interpretation of the CRT [27] needs a very high precision of $\left\lceil \log_{2}\left(kM_{k}\right)\right\rceil$ bits. The method proposed in [28,29] uses a sequential bit-by-bit manner for evaluating reconstruction coefficient *r*. The iterative structure of this method makes it very slow in the case of large word-length numbers.

There are also approaches to reconstruct the integer value of RNS number based on the CRT by using special moduli-sets with a limited number of moduli such as $m=2^{n}+d$$\left(d\in \left\{-1,0,1\right\}\right)$ [5,8]. The main drawback of these methods consists of a small number of the selected moduli, typically from three to five. Such moduli sets are suitable for the efficient implementations of digital signal processing algorithms but completely not applicable for the processing of large numbers which are widely used in cryptography.

In recent decades, the CRT algorithm, corresponding forms of number representation, and the methods of integer reconstruction by residue code have been intensively studied, especially concerning their application in high-performance computing. The major efforts are aimed at reducing the computational complexity of calculating the main integral characteristics of residue code.

There are some new approaches for calculating an approximate value of the rank of a number which allow us to reduce the computational complexity of complicated non-modular operations in RNS arithmetic [30,31,32]. The method proposed in [30] is based on the so-called interval floating-point characteristic which provides information about the range of changes in the relative value of RNS representation. Generally, it enables us to perform effectively such operations as magnitude comparison, sign determination, and overflow detection. The concept of an approximate value of the rank of a number is introduced in [31]. This approach allows us to reduce the computational complexity of the decoding from residue code to binary representation and decrease the size of the required coefficients. Based on the properties of the approximate value and arithmetic properties of RNS, a new method for error detection, correction, and controlling computational results has been proposed. In [32], a new original general-purpose technique for CRT basis extension and scaling in RNS using floating-point arithmetic for the rank estimation is proposed for a homomorphic encryption scheme. The main algorithmic improvements focus on optimizing decryption and homomorphic multiplication in the RNS using the CRT to represent and manipulate the large coefficients in the ciphertext polynomials.

The rank positional characteristic has been thoroughly investigated in [33,34]. As shown, the rank $\rho_{k}\left(X\right)$ has a simple structure, high modularity of calculation, and a small range of changes. At the same time, the rank $\rho_{k}\left(X\right)$ is a sum of two small numbers, namely, the inexact rank ${\hat{\rho }}_{k}\left(X\right)<k$ and two-valued rank correction $\Delta_{k}\left(X\right)\in \left\{0,1\right\}$:

(11) $$\rho_{k}\left(X\right)={\hat{\rho }}_{k}\left(X\right)+\Delta_{k}\left(X\right),$$

where

(12) $${\hat{\rho }}_{k}\left(X\right)=\left\lfloor \frac{1}{m_{k}}\sum_{i=1}^{k}R_{i,k}\left(\chi_{i}\right)\right\rfloor$$

and

(13) $$R_{i,k}\left(\chi_{i}\right)=\left\lfloor \frac{m_{k}\chi_{i,k}}{m_{i}}\right\rfloor =\left\lfloor \frac{m_{k}{\left|\mu_{i,k}\chi_{i}\right|}_{m_{i}}}{m_{i}}\right\rfloor \left(i=1,2,...,k-1\right),$$

(14) $$R_{k,k}\left(\chi_{k}\right)=\chi_{k,k}={\left|\mu_{k,k}\chi_{k}\right|}_{m_{k}}.$$

In conventional non-redundant RNS, as it follows from (11)–(14), the calculation of the inexact rank ${\hat{\rho }}_{k}\left(X\right)$ is reduced to a summation of *k* small residues $R_{1,k}\left(\chi_{1}\right)$, $R_{2,k}\left(\chi_{2}\right)$, …, $R_{k,k}\left(\chi_{k}\right)$ modulo $m_{k}$ taking into account the number of the overflows occurring during the modular addition operations. At the same time, as demonstrated in [34], the main computational cost is associated with the estimation of the rank correction $\Delta_{k}\left(X\right)$. Its evaluation requires concurred modular addition operations in all independent modular channels corresponding to primary RNS moduli $m_{1},m_{2},\ldots ,m_{k}$. These computations can be implemented easily by the pre-computation and lookup table techniques. As a result, the total number of required modular addition operations and lookup tables for rank $\rho_{k}\left(X\right)$ calculation are $\left(k^{2}+5k-10\right)/2$ and $\left(k^{2}+k-2\right)/2$, respectively.

As shown in [34], the minimum redundancy residue code enables optimization of the rank calculation. It assumes the extension of non-redundant residue code $\left(\chi_{1},\chi_{2},\ldots ,\chi_{k}\right)$ of the number *X* by the redundant residue ${\chi_{0}=\left|X\right|}_{m_{0}}$ concerning extra modulus $m_{0}=2$, i.e., by adding the parity of the number *X* to its residue representation. Therefore, in minimally redundant RNS, the number $X\in Z_{M_{k}}$ is represented by its minimally redundant residue code $\left(\chi_{0},\chi_{1},\ldots ,\chi_{k}\right)$. So, the total residue code length increases by only one bit.

The main advantage of minimally redundant RNS compared with non-redundant analogs consists of a significant simplification of calculating the rank correction $\Delta_{k}\left(X\right)$ and, accordingly, the rank $\rho_{k}\left(X\right)$.

The use of minimum redundancy residue code makes it possible to replace in (11) the rank correction $\Delta_{k}\left(X\right)$, which evaluation is time-consuming and requires performing addition operations in all modular channels, with a trivially calculated binary attribute $\delta_{k}\left(X\right)\in \left\{0,1\right\}$. At the same time,

(15) $$\rho_{k}\left(X\right)={\hat{\rho }}_{k}\left(X\right)+\delta_{k}\left(X\right)$$

and

(16) $$\delta_{k}\left(X\right)={\left|\chi_{0}+\sum_{i=1}^{k}\psi_{i}+{\hat{\rho }}_{0}\right|}_{2},$$

where $\chi_{0}={\left|X\right|}_{2}$, $\psi_{i}={\left|\chi_{i,k}\right|}_{2}={\left|{\left|\mu_{i,k}\chi_{i}\right|}_{m_{i}}\right|}_{2}$, and ${\hat{\rho }}_{0}={\left|{\hat{\rho }}_{k}\left(X\right)\right|}_{2}$.

Compared with non-redundant analogs, the use of minimally redundant RNS enables us to reduce significantly the complexity of the rank $\rho_{k}\left(X\right)$ calculation both in terms of required modular addition operations and lookup tables. At the same time, the corresponding computational cost is *k* modular addition operations and *k* one-input lookup tables. The time complexity depends only on the number of primary RNS moduli and equals $T_{rank}=\left\lceil \log_{2}k\right\rceil$ modular clock cycles.

As shown in [34], the transition to the minimum redundant residue code enables a decrease in the computational complexity of the rank calculation from the order $O\left(k^{2}/2\right)$ to $O\left(k\right)$ concerning required modular addition operations and lookup tables. Thus, the computational complexity reduction factor increases with the number *k* of non-redundant moduli $m_{1},m_{2},\ldots ,m_{k}$ and asymptotically approaches the threshold k/2.

The use of minimally redundant RNS ensures significant optimization of calculating the rank $\rho_{k}\left(X\right)$ of the number *X*. Moreover, this is also applied to the implementation of the CRT algorithm and, correspondingly, to the execution of various non-modular operations based on it. First of all, that is caused owing to the extreme simplicity evaluation of two-valued characteristic $\delta_{k}\left(X\right)\in \left\{0,1\right\}$ as well as the modular structure of the main calculation equation for inexact rank ${\hat{\rho }}_{k}\left(X\right)$ (see (12)). This circumstance enables radical simplifying the calculation of the rank $\rho_{k}\left(X\right)$ in minimally redundant RNS in comparison with conventional non-redundant RNS and, consequently, makes it possible to construct faster and optimal with respect to computational complexity variants of RNS arithmetic.

Therefore, the application of minimally redundant residue representation takes priority over conventional non-redundant RNS arithmetic to implement the scaling procedures based on the rank form of a number.

## 4. The Main Types of Scaling Algorithms in RNS Arithmetic

In the conventional WNS, the power-of-two scaling is performed simply by right shifting. In the RNS, compared with WNS, this procedure has substantial difficulty because it is not easily implementable due to its non-positional nature.

The classical power-of-two scaling method consists of the residue code conversion to binary representation, scaling in the conventional WNS, and converting the result back to the RNS.

Unlike the WNS, the residue code does not contain explicit information about the integer value of the represented number. Therefore, in addition to its usual purpose, which consists of limiting the undesirable growth of calculation results, the scaling in RNS is also used to detect the position of integers in a particular range (i.e., to evaluate their values), rounding, and solving other similar tasks. This operation is often used in more complex non-modular procedures such as general modular division. Many different scaling algorithms, which do not require RNS-to-binary conversion, have been presented in the literature. A detailed review of the known modular scaling methods is presented in [8].

The essence of the modular scaling operation is to obtain some integer approximation $\hat{X}=\left({\hat{\chi }}_{1},{\hat{\chi }}_{2},\ldots ,{\hat{\chi }}_{k}\right)$ $\left(i=1,2,\ldots ,k\right)$ to the fraction X/S, where $X=\left(\chi_{1},\chi_{2},\ldots ,\chi_{k}\right)$ is an arbitrary element of the RNS number range $Z_{M_{k}}$, and *S* is a constant factor (scale). The fraction X/S is usually approximated by the integers ⌊X/S⌋ and ⌈X/S⌉ (⌊x⌋ and ⌈x⌉ are the floor and ceiling function of *x*, respectively).

The most important aspect of the scaling problem in RNS is to ensure the high flexibility of the created algorithmic tools. That implies adoption of the set $S=\left\{S_{0},S_{1},\ldots ,S_{\Lambda -1}\right\}$ of scales $S_{l}>1$ $\left(l=0,1,\ldots ,\Lambda -1\right)$ which is usually chosen based on the criterion for the minimum calculating error under a given constraint on the number of scaling factors.

All known scaling techniques can be classified into four main categories:

1. scaling by the product of some RNS moduli [32,35,36,37,38],
2. scaling by an integer from the RNS number range [39,40],
3. scaling by a common fraction [41],
4. scaling by a power of two [42,43,44].

In the first group, many scaling methods take the scaling factor *S* as a product of *l* moduli, i.e., of the form $S=M_{l}$ $\left(l=1,2,\ldots ,k-1\right)$ [35,36,37,38]. That makes it easier to obtain the residues ${\hat{\chi }}_{l+1},{\hat{\chi }}_{l+2},\ldots ,{\hat{\chi }}_{k}$ of the approximation $\hat{X}$ to the fraction X/S. The remaining residues ${\hat{\chi }}_{1},{\hat{\chi }}_{2},\ldots ,{\hat{\chi }}_{l}$ can be calculated sufficiently lightly within the framework of procedures based on one of the base extension algorithms [2,35,45]. Due to the small word length of residues, the pre-computation and lookup table techniques are suitable for modular scaling.

In [35], the base extension algorithm uses the reverse conversion of residue code to mixed-radix representation. The method proposed in [36] requires a redundant modulus to evaluate the CRT reconstruction coefficient, i.e., the rank of a number, to complete the base extension procedure. In [38], the suggested approach is entirely based on a lookup tables technique, while all the required tables have two inputs. At the same time, the memory costs are too high when the number of chosen moduli is sufficiently large. The method proposed in [37] enables one to carry out base extension and exact scaling without some system redundancy only by using additional lookup tables.

The CRT-base technique for modular scaling by an integer has been suggested in [39]. Here, the main idea is to approximate the CRT calculating relation for reconstructing the integer value of RNS numbers. This enables the substitution of large modulo $M_{k}$ addition in the canonic CRT-decoding scheme by smaller word-length modular addition operations. In [40], the proposed method uses minimum redundancy for modular scaling by arbitrary positive scales. The distinctive feature of the algorithm consists of using the interval index as a positional characteristic of residue code. At the same time, the interval index can be calculated fast and lightly by modular addition of small residues in the *k*th modular channel corresponding to the modulus $m_{k}$ from the RNS moduli-set $\left\{m_{1},m_{2},\ldots ,m_{k}\right\}$.

In the case of arbitrary rational scale *S*, an efficient basis for modular scaling is the approach presented in [41]. The main feature is that for the scales of the form S=p/q, the numbers *p* and *q* can take any integer values for which the fraction qX/p does not exceed the upper bound of the RNS number range. In addition, both the number qX and the results of intermediate calculations may not satisfy the specified requirement.

The scaling methods in the fourth group implement division by constants of the form $S=2^{l}$ $\left(l=1,2,\ldots ,\Lambda \right)$, $\Lambda \leq \lfloor \log_{2}M_{k}\rfloor$ [7,42,43]. General approaches to solving this task are based mainly on the bisection method. It consists of calculating the recurrence relation $X^{\left(j+1\right)}=\left\lfloor X^{\left(j\right)}/2\right\rfloor$ for j=0,1,…,l−1. In this case, $X^{\left(0\right)}=X$, and $X^{\left(l\right)}=\left\lfloor X/2^{l}\right\rfloor$. The residue ${\hat{\chi }}_{i}^{\left(j+1\right)}$ $\left(i=1,2,\ldots ,k\right)$ of approximation $X^{\left(j+1\right)}$ is determined as

(17) $$\chi_{i}^{\left(j+1\right)}=\left\{\begin{array}{c} {\left|\frac{1}{2}\chi_{i}^{\left(j\right)}\right|}_{m_{i}}\;\;\mathrm{if}X^{\left(j\right)}\mathrm{is}\mathrm{even},\\ {\left|\frac{1}{2}\left(\chi_{i}^{\left(j\right)}-1\right)\right|}_{m_{i}}\;\;\mathrm{if}X^{\left(j\right)}\mathrm{is}\mathrm{odd}, \end{array}\right.$$

while all the primary moduli $m_{1},m_{2},\ldots ,m_{k}$ are coprime odd numbers. The last condition ensures that 2 and $m_{i}$(i=1,2,…,k) are relatively prime numbers, and, correspondingly, the existence of a modular multiplicative inverse of 2, i.e., the number ${\left|2^{-1}\right|}_{M_{k}}=\left({\left|2^{-1}\right|}_{m_{1}},{\left|2^{-1}\right|}_{m_{2}},\ldots ,{\left|2^{-1}\right|}_{m_{k}}\right)$. As followed from (17), the scaling by 2 requires the parity detection of the number $X^{\left(j\right)}$, j=0,1,…,l−1. So, there is a need for a base extension operation to extra modulus equal 2.

An iterative algorithm for scaling by the factor $S=2^{l}$ proposed in [42] is implemented in *l* steps. At the same time, the parity of the intermediate results is checked at each iteration using the base extension operation suggested in [25]. In [43], the power-of-two scaling technique is applied to realize a digital filter in quadratic RNS. The scaling algorithm presented in [44] focuses on arbitrary moduli sets with large dynamic ranges and requires only machine-precision integer and floating-point operations. At the same time, it is used for software implementation of rounding and exponent alignment procedures in a multiple-precision RNS-based arithmetic library for parallel CPU-GPU systems.

Many modular scaling algorithms use special moduli sets with a limited number of moduli. A detailed review of some of these methods is given in [8]. The most commonly used moduli sets for efficient RNS scalers are $\left\{2^{2n+1}+1,2^{2n+1},2^{2n+1}-1\right\}$, $\left\{2^{n}-1,2^{n+p},2^{n}+1\right\}$, $\left\{2^{n+1}-1,2^{n},2^{n}-1\right\}$, $\left\{2^{n+p},2^{n}-1,2^{n-1}-1\right\}$ among others [46,47,48,49,50,51]. The main drawback of such approaches is imposing very restrictive constraints on the moduli sets. They are certainly suitable for implementing scaling tasks in digital signal processing but, at the same time, they do not fit for scaling and other non-modular operations on numbers belonging to large dynamic ranges which are widely used in long-word-length cryptography.

## 5. A Novel Approach for Calculating the Rank of a Number Resulting from Scaling by 2

In RNS, the rank $\rho_{k}\left(X\right)\in Z_{k}=\left\{0,1,\ldots ,k-1\right\}$ is a principal positional characteristic since all the non-modular operations, such as magnitude comparison, sign determination, overflow detection, general division, scaling, residue-to-binary conversion, and others, can be implemented on its basis. Because the rank $\rho_{k}\left(X\right)$ enables estimation of the integer value of the RNS-number *X*, then the development of efficient methods and algorithms for its calculating is of primary importance in building efficient variants of RNS arithmetic and, accordingly, high-performance modular computational structures.

Let us show that the rank form (9) of the number representation in residue arithmetic creates a basis for constructing relatively fast and sufficiently simple iterative algorithms for the implementation of division by constant $S_{l}=2^{l}$$\left(l=1,2,\ldots ,\Lambda ,\Lambda \leq \lfloor \log_{2}M_{k}\rfloor \right)$. In this case, the following theorem is fundamental for solving the problem of modular scaling by powers of 2.

**Theorem** **2.** *Let in RNS with pairwise prime odd moduli $m_{1},m_{2},\ldots ,m_{k}$ the arbitrary number $X=\left(\chi_{1},\chi_{2},\ldots ,\chi_{k}\right)$ from the range $Z_{M_{k}}$ having rank $\rho_{k}\left(X\right)$ be given. Then the rank of the integer $\hat{X}=\left\lfloor X/2\right\rfloor$ satisfies the equation*

(18) $$\rho_{k}\left(\hat{X}\right)=\left\{\begin{array}{c} \frac{1}{2}\left(\rho_{k}\left(X\right)+\sum_{i=1}^{k}\psi_{i}\right)\;\;\mathrm{if}X\mathrm{is}\mathrm{even},\\ \frac{1}{2}\left(\rho_{k}\left(X\right)-\rho_{k}\left(1\right)+\sum_{i=1}^{k}\omega_{i}+\sum_{i=1}^{k}\varphi_{i}\right)\;\;\mathrm{if}X\mathrm{is}\mathrm{odd}, \end{array}\right.$$

*where*

(19) $$\psi_{i}={\left|\chi_{i,k}\right|}_{2}={\left|{\left|\mu_{i,k}\chi_{i}\right|}_{m_{i}}\right|}_{2},$$

(20) $$\omega_{i}=\left\{\begin{array}{c} 0\;\;\mathrm{if}\;\;\chi_{i,k}\geq \mu_{i,k},\\ 1\;\;\mathrm{if}\;\;\chi_{i,k}<\mu_{i,k}, \end{array}\right.$$

(21) $$\varphi_{i}=\left\{\begin{array}{c} {\left|\psi_{i}+\omega_{i}\right|}_{2}\;\;\mathrm{if}\;{\left|\mu_{i,k}\right|}_{2}=0,\\ \bar{{\left|\psi_{i}+\omega_{i}\right|}_{2}}\;\;\mathrm{if}\;{\left|\mu_{i,k}\right|}_{2}=1, \end{array}\right.$$

*$\rho_{k}\left(1\right)$ is the rank of the number *1*, and $\bar{x}$ denotes the negation of the Boolean value x.*

**Proof.**  As follows from the rank form (9), the number 1 in a given RNS has the following form

$$1=\sum_{i=1}^{k}M_{i,k}\mu_{i,k}-\rho_{k}\left(1\right)M_{k}.$$

Therefore, we can write

$$\hat{X}=\left\lfloor X/2\right\rfloor =\frac{1}{2}\left(X-{\left|X\right|}_{2}\right)=$$

$$=\frac{1}{2}\left(\sum_{i=1}^{k}M_{i,k}\chi_{i,k}-\rho_{k}\left(X\right)M_{k}-{\left|X\right|}_{2}\left(\sum_{i=1}^{k}M_{i,k}\mu_{i,k}-\rho_{k}\left(1\right)M_{k}\right)\right)=$$

(22) $$=\frac{1}{2}\left(\sum_{i=1}^{k}M_{i,k}\left(\chi_{i,k}-{\left|X\right|}_{2}\mu_{i,k}\right)-M_{k}\left(\rho_{k}\left(X\right)-{\left|X\right|}_{2}\rho_{k}\left(1\right)\right)\right).$$

Then, in accordance with Euclid’s Division Lemma (1), from (22) we have

$$\begin{array}{c} \hat{X}=\frac{1}{2}\left(\sum_{i=1}^{k}M_{i,k}\left({\left|\chi_{i,k}-{\left|X\right|}_{2}\mu_{i,k}\right|}_{m_{i}}+\left\lfloor \left(\chi_{i,k}-{\left|X\right|}_{2}\mu_{i,k}\right)/m_{i}\right\rfloor m_{i}\right)-\right.\\ \left.-M_{k}\left(\rho_{k}\left(X\right)-{\left|X\right|}_{2}\rho_{k}\left(1\right)\right)\right). \end{array}$$

Thus,

(23) $$\begin{array}{c} \hat{X}=\sum_{i=1}^{k}\frac{1}{2}M_{i,k}{\left|\chi_{i,k}-{\left|X\right|}_{2}\mu_{i,k}\right|}_{m_{i}}-\frac{1}{2}M_{k}\left(\rho_{k}\left(X\right)-{\left|X\right|}_{2}\rho_{k}\left(1\right)-\right.\\ \left.-\sum_{i=1}^{k}\left\lfloor \left(\chi_{i,k}-{\left|X\right|}_{2}\mu_{i,k}\right)/m_{i}\right\rfloor \right). \end{array}$$

Since for each least nonnegative residue $\chi \in Z_{m}$ modulo an arbitrary odd modulus *m*, there is a unique formal quotient ${\left|\chi /2\right|}_{m}$, and

$${\left|\chi /2\right|}_{m}=\left(\chi +m{\left|\chi \right|}_{2}\right)/2$$

(see, for example, [1]), then

$${\hat{\chi }}_{i,k}={\left|\frac{1}{2}{\left|\chi_{i,k}-{\left|X\right|}_{2}\mu_{i,k}\right|}_{m_{i}}\right|}_{m_{i}}=\frac{1}{2}\left({\left|\chi_{i,k}-{\left|X\right|}_{2}\mu_{i,k}\right|}_{m_{i}}+m_{i}{\left|{\left|\chi_{i,k}-{\left|X\right|}_{2}\mu_{i,k}\right|}_{m_{i}}\right|}_{2}\right).$$

Therefore,

$$\frac{1}{2}{\left|\chi_{i,k}-{\left|X\right|}_{2}\mu_{i,k}\right|}_{m_{i}}={\hat{\chi }}_{i,k}-\frac{1}{2}m_{i}{\left|{\left|\chi_{i,k}-{\left|X\right|}_{2}\mu_{i,k}\right|}_{m_{i}}\right|}_{2}.$$

Taking this into account, from (23) we get

$$\begin{array}{c} \hat{X}=\sum_{i=1}^{k}M_{i,k}{\hat{\chi }}_{i,k}-\frac{1}{2}\left(\rho_{k}\left(X\right)-{\left|X\right|}_{2}\rho_{k}\left(1\right)-\sum_{i=1}^{k}\left\lfloor \left(\chi_{i,k}-{\left|X\right|}_{2}\mu_{i,k}\right)/m_{i}\right\rfloor +\right.\\ \left.+\sum_{i=1}^{k}{\left|{\left|\chi_{i,k}-{\left|X\right|}_{2}\mu_{i,k}\right|}_{m_{i}}\right|}_{2}\right). \end{array}$$

Hence, according to the rank form of number representation (9), we conclude that the following equation for the rank $\rho_{k}\left(\hat{X}\right)$ of the number $\hat{X}$ is valid:

(24) $$\begin{array}{c} \rho_{k}\left(\hat{X}\right)=\frac{1}{2}\left(\rho_{k}\left(X\right)-{\left|X\right|}_{2}\rho_{k}\left(1\right)-\sum_{i=1}^{k}\left\lfloor \left(\chi_{i,k}-{\left|X\right|}_{2}\mu_{i,k}\right)/m_{i}\right\rfloor +\right.\\ \left.+\sum_{i=1}^{k}{\left|{\left|\chi_{i,k}-{\left|X\right|}_{2}\mu_{i,k}\right|}_{m_{i}}\right|}_{2}\right). \end{array}$$

If the number *X* is even, then ${\left|X\right|}_{2}=0$, so that

$$\left\lfloor \left(\chi_{i,k}-{\left|X\right|}_{2}\mu_{i,k}\right)/m_{i}\right\rfloor =\left\lfloor \chi_{i,k}/m_{i}\right\rfloor =0$$

and

$${\left|{\left|\chi_{i,k}-{\left|X\right|}_{2}\mu_{i,k}\right|}_{m_{i}}\right|}_{2}={\left|\chi_{i,k}\right|}_{2}=\psi_{i}$$

$\left(i=1,2,\ldots ,k\right)$. Therefore, in this case, Equation (24) takes the form

$$\rho_{k}\left(\hat{X}\right)=\frac{1}{2}\left(\rho_{k}\left(X\right)+\sum_{i=1}^{k}\psi_{i}\right)$$

which corresponds to (18).

If the number *X* is odd, then ${\left|X\right|}_{2}=1$, and it is easy to check that

$$\left\lfloor \left(\chi_{i,k}-{\left|X\right|}_{2}\mu_{i,k}\right)/m_{i}\right\rfloor =\left\lfloor \left(\chi_{i,k}-\mu_{i,k}\right)/m_{i}\right\rfloor =-\omega_{i},$$

while

$${\left|{\left|\chi_{i,k}-{\left|X\right|}_{2}\mu_{i,k}\right|}_{m_{i}}\right|}_{2}={\left|{\left|\chi_{i,k}-\mu_{i,k}\right|}_{m_{i}}\right|}_{2}=\varphi_{i}$$

$\left(i=1,2,\ldots ,k\right)$, where $\omega_{i}$ and $\varphi_{i}$ are two-valued quantities determined by (19) and (20), respectively. In this case, Equation (24) takes the form

$$\rho_{k}\left(\hat{X}\right)=\frac{1}{2}\left(\rho_{k}\left(X\right)-\rho_{k}\left(1\right)+\sum_{i=1}^{k}\omega_{i}+\sum_{i=1}^{k}\varphi_{i}\right)$$

which also corresponds to (18).

The theorem is proved.    □

As it follows from Theorem 2, the rank $\rho_{k}\left(\hat{X}\right)$ of the number $\hat{X}=\left\lfloor X/2\right\rfloor$ can be calculated rapidly and easily only taking into account the known value of the rank $\rho_{k}\left(X\right)$ of the initial number *X*. This circumstance makes it possible to optimize and significantly speed up the execution of the power-of-two scaling operation. In this case, it is not necessary at each iteration to calculate the rank of the number, which is the intermediate result of scaling, by its residue code. At the same time, the complete operation of rank calculation is necessary only for the initial number *X* at the preliminary stage of the scaling procedure.

## 6. A Novel Power-of-Two Modular Scaling Based on the Rank Positional Characteristic in Minimally Redundant RNS

Theorem 2 implies the following step algorithm for power-of-two scaling in minimally redundant RNS with primary pairwise prime odd modules $m_{1},m_{2},\ldots ,m_{k}$, extra modulus $m_{0}=2$, and scales of the form $S_{l}=2^{l}$$\left(l=1,2,\ldots ,\Lambda ,\Lambda =\lfloor \log_{2}M_{k}\rfloor \right)$.

**S.1.** Based on the minimum redundant residue code $\left(\chi_{0},\chi_{1},\ldots ,\chi_{k}\right)$ of the original number *X*, the rank $\rho_{k}\left(X\right)$ is calculated following to (12)–(16). In addition, it is assumed that $X^{\left(0\right)}=X$, $\chi_{i}^{\left(0\right)}=\chi_{i}$$\left(i=0,1,\ldots ,k\right)$, $\chi_{i,k}^{\left(0\right)}=\chi_{i,k}={\left|\mu_{i,k}\chi_{i}\right|}_{m_{i}}$$\left(i=1,2,\ldots ,k\right)$, and j=0.

**S.2.** For the residue number $X^{\left(j\right)}=\left(\chi_{0}^{\left(j\right)},\chi_{1}^{\left(j\right)},\ldots ,\chi_{k}^{\left(j\right)}\right)$, the integer

(25) $$\Delta^{\left(j\right)}=\left\{\begin{array}{c} \sum_{i=1}^{k}\psi_{i}^{\left(j\right)}\;\;\mathrm{if}\;\;\chi_{0}^{\left(j\right)}=0,\\ \sum_{i=1}^{k}\omega_{i}^{\left(j\right)}+\sum_{i=1}^{k}\varphi_{i}^{\left(j\right)}-\rho_{k}\left(1\right)\;\;\mathrm{if}\;\;\chi_{0}^{\left(j\right)}=1 \end{array}\right.$$

is calculated, where

(26) $$\psi_{i}^{\left(j\right)}={\left|\chi_{i,k}^{\left(j\right)}\right|}_{2},$$

$\omega_{i}^{\left(j\right)}$ and $\varphi_{i}^{\left(j\right)}$ are obtained by formulas similar to (19) and (20), namely:

(27) $$\omega_{i}^{\left(j\right)}=\left\{\begin{array}{c} 0\;\;\mathrm{if}\;\;\chi_{i,k}^{\left(j\right)}\geq \mu_{i,k},\\ 1\;\;\mathrm{if}\;\;\chi_{i,k}^{\left(j\right)}<\mu_{i,k}, \end{array}\right.$$

(28) $$\varphi_{i}^{\left(j\right)}=\left\{\begin{array}{c} {\left|\psi_{i}^{\left(j\right)}+\omega_{i}^{\left(j\right)}\right|}_{2}\;\;\mathrm{if}\;\;{\left|\mu_{i,k}\right|}_{2}=0,\\ \bar{{\left|\psi_{i}^{\left(j\right)}+\omega_{i}^{\left(j\right)}\right|}_{2}}\;\;\mathrm{if}\;\;{\left|\mu_{i,k}\right|}_{2}=1, \end{array}\right.$$

i=1,2,…,k.

**S.3.** The digits $\chi_{1}^{\left(j+1\right)}$, $\chi_{2}^{\left(j+1\right)}$, ⋯, $\chi_{k}^{\left(j+1\right)}$ of the minimally redundant residue code and the rank $\rho_{k}\left(X^{\left(j+1\right)}\right)$ of the number $X^{\left(j+1\right)}=\left\lfloor X^{\left(j\right)}/2\right\rfloor$ are determined, respectively, according to the rules

(29) $$\chi_{i}^{\left(j+1\right)}={\left|\frac{1}{2}\left(\chi_{i}^{\left(j\right)}-\chi_{0}^{\left(j\right)}\right)\right|}_{m_{i}}\;\left(i=1,2,\ldots ,k\right),$$

(30) $$\rho_{k}\left(X^{\left(j+1\right)}\right)=\frac{1}{2}\left(\rho_{k}\left(X^{\left(j\right)}\right)+\Delta^{\left(j\right)}\right).$$

**S.4.** The redundant residue $\chi_{0}^{\left(j+1\right)}={\left|(X^{\left(j+1\right)}\right|}_{2}$ is calculated according to equation following from the rank form (9)

(31) $$\chi_{0}^{\left(j+1\right)}={\left|\sum_{i=1}^{k}\psi_{i}^{\left(j+1\right)}+\rho_{0}^{\left(j+1\right)}\right|}_{2},$$

where $\psi_{i}^{\left(j+1\right)}={\left|\chi_{i,k}^{\left(j+1\right)}\right|}_{2}={\left|{\left|\mu_{i,k}\chi_{i}^{\left(j+1\right)}\right|}_{m_{i}}\right|}_{2}$ and $\rho_{0}^{\left(j+1\right)}={\left|\rho_{k}\left(X^{\left(j+1\right)}\right)\right|}_{2}$. In essence, it determines the parity of the number $X^{\left(j+1\right)}$.

If j=l−1, then the number $X^{\left(j+1\right)}=X^{\left(l\right)}=\left\lfloor X/2^{l}\right\rfloor$ is the required number, and the scaling process ends. Otherwise, the variable *j* is incremented by one (j=j+1), and the jump to step S.2 is carried out.

For its hardware implementation, the most important feature of the above recursive scaling algorithm is that the specified operations on steps S.2, S.3, and S.4 can be combined in time and carried out within one modular clock cycle. Due to this circumstance, after obtaining the rank $\rho_{k}\left(X\right)$, each iteration of RNS number scaling by 2, i.e., each shift of its integer value by one bit to the right, is performed in one modular clock cycle.

Since the calculating process of the rank $\rho_{k}\left(X\right)$ has a pipeline structure, with the appropriate organization of computations the described scaling procedure at low hardware costs provides a reasonably high speed.

It follows from the above that all the necessary calculations within the scaling algorithm can be implemented using tabular computational structures.

For example, the calculation of the inexact rank ${\hat{\rho }}_{k}\left(X\right)$ of the initial number *X* is reduced to a summation of the sets of small residues $\langle R_{1,k}\left(\chi_{1}\right),R_{2,k}\left(\chi_{2}\right),\ldots ,R_{k,K}\left(\chi_{k}\right)\rangle$ modulo $m_{k}$. Simultaneously, we take into account the number of occurred overflows when performing these modular addition operations (see (12)–(14)). Therefore, we need *k* one-input lookup tables to store the given set, while the bit length of recorded residues is $\left\lceil \log_{2}m_{k}\right\rceil \left(l=1,2,\ldots ,k\right)$. At the same time, the estimation of two-valued rank correction $\delta_{k}\left(X\right)$ (see (16)) requires the set $\langle \psi_{1},\psi_{2},\ldots ,\psi_{k}\rangle$ of least significant bits of normalized residues $\chi_{i,k}$$\left(i=1,2,\ldots ,k\right)$ of the number *X* (see (6)).

Similarly, the sets of binary flags $\langle \psi_{1}^{\left(j\right)},\psi_{2}^{\left(j\right)},\ldots ,\psi_{k}^{\left(j\right)}\rangle$, $\langle \omega_{1}^{\left(j\right)},\omega_{2}^{\left(j\right)},\ldots ,\omega_{k}^{\left(j\right)}\rangle$ and also $\langle \varphi_{1}^{\left(j\right)},\varphi_{2}^{\left(j\right)},\ldots ,\varphi_{k}^{\left(j\right)}\rangle$ (see (26)–(28)) enable us to obtain the integer $\Delta^{\left(j\right)}$ required for rank calculating in the corresponding iterations of scaling procedure $\left(j=0,1,\ldots ,l-1\right)$. All these binary sets can also be recorded in the appropriate lookup tables.

Thus, the content of the *i*th lookup table corresponding to the input residue $\chi_{i}^{\left(j\right)}$ has the form $\langle R_{i,k}\left(\chi_{i}^{\left(j\right)}\right),\psi_{i}^{\left(j\right)},\omega_{i}^{\left(j\right)},\varphi_{i}^{\left(j\right)}\rangle$ $\left(i=1,2,\ldots ,k\right),\left(j=0,1,\ldots ,l-1\right)$.

Below we present the proposed scaling method in the form of a pseudo-code algorithm.

Let us evaluate the computational complexity of the proposed iterative power-of-two scaling method. As follows from the above, Algorithm 1 requires total $T_{scal}=T_{rank}+T_{iter}\times l$ modular clock cycles. According to [33,34], in minimally redundant RNS, the time complexity of calculating the rank $\rho_{k}\left(X\right)$ of the initial number *X* depends only on the number *k* of primary RNS moduli and can be evaluated as $T_{rank}=\left\lceil \log_{2}k\right\rceil$. At the same time, all calculations within each iteration, consisting in obtaining both the minimally redundant residue code $\left(\chi_{0}^{\left(j+1\right)},\chi_{1}^{\left(J+1\right)},\ldots ,\chi_{k}^{\left(j+1\right)}\right)$ and the rank $\rho_{k}\left(X^{\left(j+1\right)}\right)$ of the number $X^{\left(j+1\right)}=\left\lfloor X^{\left(j\right)}/2\right\rfloor$$\left(j=0,1,\ldots ,l-1\right)$, can be performed in one modular clock cycle by using lookup table technique. Therefore, $T_{iter}=1$. Hence, the algorithm time complexity $T_{scal}=\left\lceil \log_{2}k\right\rceil +l$ modular clock cycles.   
**Algorithm 1:** Power-of-two scaling in minimally redundant RNS
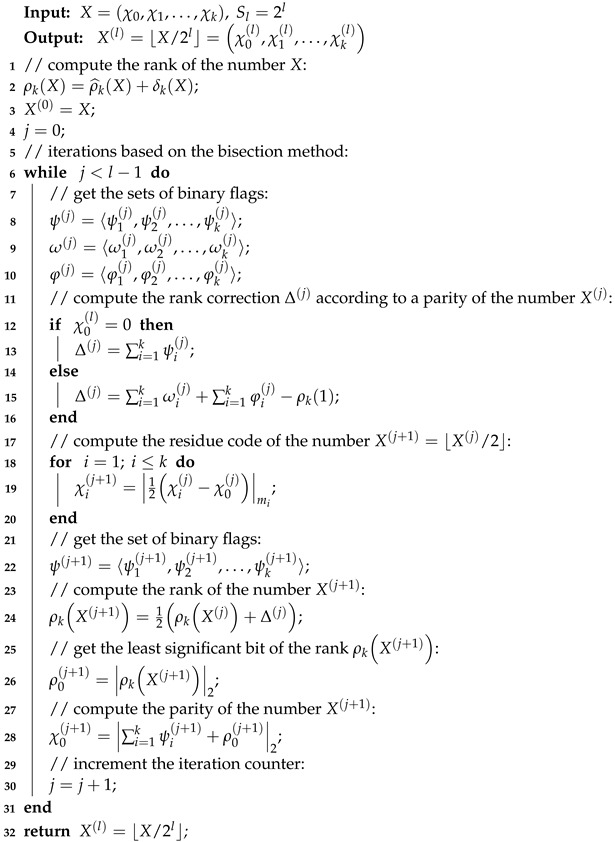


To illustrate the power-of-two scaling of the number $X=\left(\chi_{0},\chi_{1},\ldots ,\chi_{k}\right)$ based on the rank form (9) in the proposed minimally redundant RNS, we present below a numerical example.

Let us consider the RNS with the primary moduli $m_{1}=5$, $m_{2}=7$, $m_{3}=9$, and $m_{4}=11$, taking into account the excess modulus $m_{0}=2.$

**Example** **1.** *Suppose we wish to scale the number X=1731 having the minimally redundant residue code $\left(\chi_{0},\chi_{1},\chi_{2},\chi_{3}\chi_{4}\right)=\left(1,1,2,3,4\right)$ by the constant $S_{3}=2^{3}=8$.*

*Therefore, the number of required iterations is l=3.*

*Before describing the proposed scaling algorithm, we give below the required primitive constants used in the RNS under consideration. So, we have*

*$M_{4}=3465$,*

*$M_{1,4}=693,M_{2,4}=495,M_{3,4}=385,M_{4,4}=315$,*

*$\mu_{1,4}=2$, $\mu_{2,4}=3$, $\mu_{3,4}=4$, $\mu_{4,4}=8$,*

*$\rho_{4}\left(1\right)=2$.*

***S.1. The rank calculation of the initial number.***

*First, having the non-redundant residue code $\left(1,2,3,4\right)$ of the number X, by using lookup tables, we obtain the following sets of residues and least-significant bits, respectively,*

*$\langle R_{1,4}\left(\chi_{1}\right),R_{2,4}\left(\chi_{2}\right),R_{3,4}\left(\chi_{3}\right),R_{4,4}\left(\chi_{4}\right)\rangle =\langle 4,9,3,10\rangle$,*

*$\langle \psi_{1},\psi_{2},\psi_{3},\psi_{4}\rangle =\langle 0,0,1,0\rangle$.*

*Let us show in more detail how these values were obtained, according to *(6)*, *(19)*, and *(13)*, *(14)*, respectively, before storing in the lookup tables:*

*$\chi_{1,4}={\left|2\cdot 1\right|}_{5}=2$, $\psi_{1}={\left|2\right|}_{2}=0$,*

*$\chi_{2,4}={\left|3\cdot 2\right|}_{7}=6$, $\psi_{2}={\left|6\right|}_{2}=0$,*

*$\chi_{3,4}={\left|4\cdot 3\right|}_{9}=3$, $\psi_{3}={\left|3\right|}_{2}=1$,*

*$\chi_{4,4}={\left|8\cdot 4\right|}_{11}=10$, $\psi_{4}={\left|10\right|}_{2}=0$,*

*$R_{1,4}\left(\chi_{1}\right)=\left\lfloor \left(11\cdot 2\right)/5\right\rfloor =4$,*

*$R_{2,4}\left(\chi_{2}\right)=\left\lfloor \left(11\cdot 6\right)/7\right\rfloor =9$,*

*$R_{3,4}\left(\chi_{3}\right)=\left\lfloor \left(11\cdot 3\right)/9\right\rfloor =3$,*

*$R_{4,4}\left(\chi_{4}\right)={\left|8\cdot 4\right|}_{11}=10$.*

*Further, using the set of residues 〈4,9,3,10〉, according to *(12)*, we calculate the inexact rank*

$${\hat{\rho }}_{4}\left(X\right)=\left\lfloor \left(4+9+3+10\right)/11\right\rfloor =\left\lfloor 26/11\right\rfloor =2,$$

*and also take its parity bit*

$${\hat{\rho }}_{0}={\left|{\hat{\rho }}_{4}\left(X\right)\right|}_{2}=0.$$

*Then, taking into account that $\chi_{0}=1$, using the set $\langle \psi_{1},\psi_{2},\psi_{3},\psi_{4}\rangle =\langle 0,0,1,0\rangle$ and ${\hat{\rho }}_{0}$, according to *(16)*, we find two-valued correction*

$$\delta_{4}\left(X\right)={\left|1+\left(0+0+1+0\right)+0\right|}_{2}={\left|2\right|}_{2}=0.$$

*As a result, according to *(16)*, we get the exact rank of the initial number X*

$$\rho_{4}\left(X\right)={\hat{\rho }}_{4}\left(X\right)+\delta_{4}\left(X\right)=2+0=2.$$

*To verify the obtained result, using the rank form *(9)*, we find*

$$X=\sum_{i=1}^{4}M_{i,4}\chi_{i,4}-\rho_{4}\left(X\right)M_{4}=693\cdot 2+495\cdot 6+385\cdot 3+315\cdot 10-2\cdot 3465=1731.$$

*In addition, it is assumed that j=0, $X^{\left(0\right)}=X$, $\chi_{i}^{\left(0\right)}=\chi_{i}$$\left(i=\bar{0,4}\right)$, $\chi_{i,4}^{\left(0\right)}=\chi_{i,4}$, $\psi_{i}^{\left(0\right)}=\psi_{i}$$\left(i=\bar{1,4}\right)$.*

***Iteration 1.***

***S.2.1.*** *Since $\chi_{0}^{\left(0\right)}=1$, using the sets of binary flags (see *(27)* and *(28)*)*

*$\langle \omega_{1}^{\left(0\right)},\omega_{2}^{\left(0\right)},\omega_{3}^{\left(0\right)},\omega_{4}^{\left(0\right)}\rangle =\langle 0,0,1,0\rangle$,*

*$\langle \varphi_{1}^{\left(0\right)},\varphi_{2}^{\left(0\right)},\varphi_{3}^{\left(0\right)},\varphi_{4}^{\left(0\right)}\rangle =\langle 0,1,0,0\rangle$,*

*according to *(25)*, we calculate the quantity*

$$\Delta^{\left(0\right)}=\sum_{i=1}^{4}\omega_{i}^{\left(0\right)}+\sum_{i=1}^{4}\varphi_{i}^{\left(0\right)}-\rho_{k}\left(1\right)=1+1-2=0.$$

***S.3.1.*** *We calculate the non-redundant residue code and the rank of the number $X^{\left(1\right)}=\left\lfloor X^{\left(0\right)}/2\right\rfloor$, according to *(29)* and *(30)*, respectively:*

*$\left(\chi_{1}^{\left(1\right)},\chi_{2}^{\left(1\right)},\chi_{3}^{\left(1\right)},\chi_{4}^{\left(1\right)}\right)=\left(0,4,1,7\right)$,*

*$\rho_{4}\left(X^{\left(1\right)}\right)=\frac{1}{2}\left(\rho_{4}\left(X^{\left(0\right)}\right)+\Delta^{\left(0\right)}\right)=\frac{1}{2}\left(2+0\right)=1$,*

*$\rho_{0}^{\left(1\right)}={\left|\rho_{4}\left(X^{\left(1\right)}\right)\right|}_{2}=1$.*

***S.4.1.*** *Using the set $\langle \psi_{1}^{\left(1\right)},\psi_{2}^{\left(1\right)},\psi_{3}^{\left(1\right)},\psi_{4}^{\left(1\right)}\rangle =\langle 0,1,0,1\rangle$ corresponding to the non-redundant residue code $\left(0,4,1,7\right)$ and taking into account that $\rho_{0}^{\left(1\right)}=1$, according to *(31)*, we find*

*$\chi_{0}^{\left(1\right)}={\left|\left(0+1+0+1\right)+1\right|}_{2}=1$.*

*Hence, as a result of Iteration 1, we have the minimally redundant residue code $\left(1,0,4,1,7\right)$ of the number $X^{\left(1\right)}=\left\lfloor X^{\left(0\right)}/2\right\rfloor =\left\lfloor 1731/2\right\rfloor =864$.*

***Iteration 2.***

***S.2.2.*** *Since $\chi_{0}^{\left(1\right)}=1$, using the following sets of binary flags*

*$\langle \omega_{1}^{\left(1\right)},\omega_{2}^{\left(1\right)},\omega_{3}^{\left(1\right)},\omega_{4}^{\left(1\right)}\rangle =\langle 1,0,0,1\rangle$,*

*$\langle \varphi_{1}^{\left(1\right)},\varphi_{2}^{\left(1\right)},\varphi_{3}^{\left(1\right)},\varphi_{4}^{\left(1\right)}\rangle =\langle 1,0,0,0\rangle$,*

*we have*

$$\Delta^{\left(1\right)}=\sum_{i=1}^{4}\omega_{i}^{\left(1\right)}+\sum_{i=1}^{4}\varphi_{i}^{\left(1\right)}-\rho_{k}\left(1\right)=2+1-2=1.$$

***S.3.2.*** *We calculate the non-redundant residue code and the rank of the number $X^{\left(2\right)}=\left\lfloor X^{\left(1\right)}/2\right\rfloor$:*

*$\left(\chi_{1}^{\left(2\right)},\chi_{2}^{\left(2\right)},\chi_{3}^{\left(2\right)},\chi_{4}^{\left(2\right)}\right)=\left(2,5,0,3\right)$,*

*$\rho_{4}\left(X^{\left(2\right)}\right)=\frac{1}{2}\left(\rho_{4}\left(X^{\left(1\right)}\right)+\Delta^{\left(1\right)}\right)=\frac{1}{2}\left(1+1\right)=1$,*

*$\rho_{0}^{\left(2\right)}={\left|\rho_{4}\left(X^{\left(2\right)}\right)\right|}_{2}=1$.*

***S.4.2.*** *Using the set $\langle \psi_{1}^{\left(2\right)},\psi_{2}^{\left(2\right)},\psi_{3}^{\left(2\right)},\psi_{4}^{\left(2\right)}\rangle =\langle 0,1,0,0\rangle$ corresponding to the non-redundant residue code $\left(2,5,0,3\right)$ and taking into account that $\rho_{0}^{\left(2\right)}=1$, we obtain*

*$\chi_{0}^{\left(2\right)}={\left|\left(0+1+0+0\right)+1\right|}_{2}=0$.*

*Hence, as a result of Iteration 2, we have the minimally redundant residue code $\left(0,2,5,0,3\right)$ of the number $X^{\left(2\right)}=\left\lfloor X^{\left(1\right)}/2\right\rfloor =\left\lfloor X^{\left(0\right)}/4\right\rfloor =\left\lfloor 1731/4\right\rfloor =432$.*

***Iteration 3.***

***S.2.3.*** *Since $\chi_{0}^{\left(2\right)}=0$, using the set*

*$\langle \psi_{1}^{\left(2\right)},\psi_{2}^{\left(2\right)},\psi_{3}^{\left(2\right)},\psi_{4}^{\left(2\right)}\rangle =\langle 0,1,0,0\rangle$,*

*according to *(25)*, we find*

$$\Delta^{\left(2\right)}=\sum_{i=1}^{4}\psi_{i}^{\left(2\right)}=1.$$

***S.3.3.*** *We calculate the non-redundant residue code and the rank of the number $X^{\left(3\right)}=\left\lfloor X^{\left(2\right)}/2\right\rfloor$:*

*$\left(\chi_{1}^{\left(3\right)},\chi_{2}^{\left(3\right)},\chi_{3}^{\left(3\right)},\chi_{4}^{\left(3\right)}\right)=\left(1,6,0,7\right)$,*

*$\rho_{4}\left(X^{\left(3\right)}\right)=\frac{1}{2}\left(\rho_{4}\left(X^{\left(2\right)}\right)+\Delta^{\left(2\right)}\right)=\frac{1}{2}\left(1+1\right)=1$,*

*$\rho_{0}^{\left(3\right)}={\left|\rho_{4}\left(X^{\left(3\right)}\right)\right|}_{2}=1$.*

***S.4.3.*** *Using the set $\langle \psi_{1}^{\left(3\right)},\psi_{2}^{\left(3\right)},\psi_{3}^{\left(3\right)},\psi_{4}^{\left(3\right)}\rangle =\langle 0,0,0,1\rangle$ corresponding to the non-redundant residue code $\left(1,6,0,7\right)$ and taking into account that $\rho_{0}^{\left(3\right)}=1$, we get*

*$\chi_{0}^{\left(3\right)}={\left|\left(0+0+0+1\right)+1\right|}_{2}=0$.*

*Hence, as a result of Iteration 3, we have the minimally redundant residue code $\left(0,1,6,0,7\right)$ of the number $X^{\left(3\right)}=\left\lfloor X^{\left(2\right)}/2\right\rfloor =\left\lfloor X^{\left(0\right)}/8\right\rfloor =\left\lfloor 1731/8\right\rfloor =216$.*

*As far as j=l−1=2, the scaling procedure ends, and the number $X^{\left(3\right)}$ is the desired solution.*

*To verify the obtained result, according to the rank form (9), we find*

$$\begin{array}{cc} X^{\left(3\right)}= & \sum_{i=1}^{4}M_{i,4}\chi_{i,4}^{\left(3\right)}-\rho_{4}\left(X^{\left(3\right)}\right)M_{4}=693\cdot 1+495\cdot 4+385\cdot 0+315\cdot 1-1\cdot 3465=\\ = & 3681-3645=216. \end{array}$$

*The result is correct.*

The above example shows that the use of minimally redundant RNS enables us to optimize and speed up the power-of-two scaling procedure compared with the conventional non-redundant RNS to a large extent. First of all, that is caused by the extreme simplicity of calculating the inexact rank ${\hat{\rho }}_{k}\left(X\right)$ and estimating two-valued characteristic $\delta_{k}\left(X\right)$ of the initial number *X* as well as by the trivial operations for obtaining the rank $\rho_{k}\left(X^{\left(j\right)}\right)$ $\left(j=0,1,\ldots ,l-1\right)$ at each iteration of the scaling procedure (see Theorem 2).

Therefore, the proposed minimally redundant residue representation takes priority over non-redundant analogs in optimization and speed-up of the scaling and other non-modular procedures based on the CRT implementation using a rank characteristic.

## 7. Discussion

Let us now discuss the theoretical and practical aspects of the approach proposed in this paper.

As followed from (17), the power-of-two scaling algorithm based on the bisection method requires the parity detection of the number $X^{\left(j\right)}$ $\left(j=0,1,\ldots ,l-1\right)$ at each iteration. Therefore, fast calculating the residue concerning extra modulus $m_{0}=2$ is a significantly important task.

In conventional non-redundant RNS, the parity detection of the number $X^{\left(j\right)}=\left(\chi_{1}^{\left(j\right)},\chi_{2}^{\left(j\right)},\ldots ,\chi_{k}^{\left(j\right)}\right)$ is usually based on estimating the integer value of $X^{\left(j\right)}$ by the use of specific positional characteristics. The generally accepted ones are the digits of mixed-radix representation, core function, the rank of a number, and interval index [1,2,3,5,8].

In RNS arithmetic, the parity check of a number refers to complicated non-modular operations requiring high computational costs. The computational complexity of this operation is comparable to the computational complexity of the reverse conversion from the residue code into the mixed-radix representation or to the calculation of the rank of a number.

Generally, in non-redundant RNS, the implementation of parallel parity check algorithm requires $O(k^{2})$ modular addition operations [33,34]. So it can become computationally expensive for large values of *k*. Thus, for efficient implementation of the power-of-two scaling algorithm based on the bisection method, one needs to speed up and optimize the RNS parity detection technique.

In this article, the proposed approach to power-of-two scaling is based on using the rank of a number as the main RNS positional characteristic. Therefore, in our case, obtaining residue modulo $m_{0}=2$ is reduced to the calculation of the rank $\rho_{k}\left(X^{\left(j\right)}\right)$ with the following use of the rank form (9).

Hence,

$$\chi_{0}^{\left(j\right)}={\left|X^{\left(j\right)}\right|}_{2}={\left|\sum_{i=1}^{k}{\left|M_{i,k}\chi_{i,k}^{\left(j\right)}\right|}_{2}+{\left|\rho_{k}\left(X^{\left(j\right)}\right)M_{k}\right|}_{2}\right|}_{2}={\left|\sum_{i=1}^{k}\psi_{i}^{\left(j\right)}+\rho_{0}^{\left(j\right)}\right|}_{2}.$$

Thus, determining the parity of a number has a computational complexity identical to the complexity of rank calculating concerning the numbers of required modular addition operations $R_{MO}$ and lookup tables $R_{LUT}$. At the same time, obtaining the residue code $\left(\chi_{1}^{\left(j+1\right)},\chi_{2}^{\left(j+1\right)},\ldots ,\chi_{k}^{\left(j+1\right)}\right)$ of the number $X^{\left(j+1\right)}=\left\lfloor X^{\left(j\right)}/2\right\rfloor$ needs *k* additional lookup tables (see (17)).

Therefore, the computational cost of the iterative procedure of scaling by $S_{l}=2^{l}$ consists of $S_{MO}=R_{MO}\times l$ modular addition operations and $S_{LUT}=R_{LUT}+k$ lookup tables, whereas the time complexity is $T_{scal}=T_{iter}\times l$ modular clock cycles, where $T_{iter}$ is a performance time of one iteration based on the bisection method.

Thus, in conventional non-redundant RNS, the computational cost of the canonical power-of-two scaling procedure based on the bisection method (17) and the rank calculation method described in [34] is estimated as

(32) $$S_{MO}=R_{MO}\times l=\frac{1}{2}\left(k^{2}+5k-10\right)\times l.$$

(33) $$S_{LUT}=R_{LUT}+k=\frac{1}{2}\left(k^{2}+3k-2\right).$$

The main advantage of the proposed approach to power-of-two scaling over the existing ones consists in the use of minimally redundant RNS and the novel method for calculating the rank of a number resulting from division by two (see Theorem 2) in each iteration of the scaling algorithm. This circumstance enables a significant reduction of the computational complexity of the scaling algorithm.

As follows from [34], the corresponding computational cost of calculating the rank $\rho_{k}\left(X\right)$ of the initial number *X* is $R_{MO}^{★}=k$ and $R_{LUT}^{★}=k$ in terms of required modular addition operations and lookup tables, respectively. Furthermore, the performance time of the rank calculation is $T_{rank}^{★}=\left\lceil \log_{2}k\right\rceil$ modular clock cycles (see Section 3).

It is important to note that all calculations at each iteration are implemented using the lookup tables technique and the simplest combinational logic circuits.

As shown above, the minimally redundant residue code of the number $X^{\left(j+1\right)}=\left\lfloor X^{\left(j\right)}/2\right\rfloor$ $\left(j=0,1,\ldots ,l-1\right)$ is yielded in only one modular clock cycle and needs the use of k+1 additional lookup tables. At the same time, the first *k* of these lookup tables are used for obtaining the residue code $\left(\chi_{1}^{\left(j+1\right)},\chi_{2}^{\left(j+1\right)},\ldots ,\chi_{k}^{\left(j+1\right)}\right)$, while the last lookup table gives us the rank $\rho_{k}\left(X^{\left(j+1\right)}\right)$ of the number $X^{\left(j+1\right)}$ (see (29) and (30)). So, at each iteration, there are no additional modular operations.

The total numbers of required modular addition operations and lookup tables are estimated, respectively, as

(34) $$S_{MO}^{★}=k$$

and

(35) $$S_{LUT}^{★}=2k+1,$$

The time complexity of the novel power-of-two scaling algorithm is $T_{scal}^{★}=T_{rank}^{★}+l=\left\lceil \log_{2}k\right\rceil +l$ modular clock cycles.

Thus, the use of minimally redundant RNS and novel approach to rank calculation at each iteration of power-of-two scaling (see Theorem 2) enables significant decrease of the computational complexity. The corresponding reduction factors of the computational complexity, in terms of the required modular addition operations (see (32) and (34)) and lookup tables (see (33) and (35)), are

(36) $$C_{MO}\left(k,l\right)=\frac{S_{MO}}{S_{MO}^{★}}=\frac{\left(k^{2}+5k-10\right)}{2k}\times l,$$

(37) $$C_{LUT}\left(k\right)=\frac{S_{LUT}}{S_{LUT}^{★}}=\frac{k^{2}+3k-2}{4k+2}.$$

Below, Table 1 and Table 2 present these reduction factors.

It should be noted that the use of the novel method for calculating the rank $\rho_{k}\left(X^{\left(j+1\right)}\right)$ of the number $X^{\left(j+1\right)}=\left\lfloor X^{\left(j\right)}/2\right\rfloor$$\left(j=0,1,\ldots ,l-1\right)$ at each iteration of the scaling procedure (see Theorem 2) in non-redundant RNS, gives us the following computational cost

(38) $$S_{MO}^{\prime }=R_{MO}=\frac{1}{2}\left(k^{2}+5k-10\right),$$

(39) $$S_{LUT}^{\prime }=R_{LUT}+\left(k+1\right)=\frac{1}{2}\left(k^{2}+3k\right).$$

Simultaneously, the time complexity is $T_{scal}^{\prime }=\left\lceil \log_{2}k\right\rceil +l+1$ modular clock cycles.

As can be seen, the reduction factors of the computational complexity of power-of-two scaling based on Theorem 2 in minimally redundant RNS compared with conventional non-redundant RNS are represented by the following fractions

(40) $$C_{MO}^{\prime }\left(k\right)=\frac{S_{MO}^{\prime }}{S_{MO}^{★}}=\frac{k^{2}+5k-10}{2k},$$

(41) $$C_{LUT}^{\prime }\left(k\right)=\frac{S_{LUT}^{\prime }}{S_{LUT}^{★}}=\frac{k^{2}+3k}{4k+2}.$$

In this case, as follows from (40), the reduction factor $C_{MO}^{\prime }\left(k\right)=C_{MO}\left(k,1\right)$ does not depend on the value $S_{l}=2^{l}$ $\left(l=0,1,\ldots ,\Lambda -1\right)$. At the same time, $C_{LUT}^{\prime }\left(k\right)\approx C_{LUT}\left(k\right)$.

The dependence of the reduction factors $C_{MO}^{\prime }\left(k\right)$ and $C_{LUT}^{\prime }\left(k\right)$ on the number of primary RNS moduli *k* is presented in Table 3.

Thus, the use of minimally redundant RNS and novel approach to calculating the rank of a number at each iterations of bisection method enables radically simplifying the carrying out of power-of-two scaling compared with conventional non-redundant RNS. This circumstance enables us to construct faster and optimal in computational cost RNS-oriented complicated computing procedures which widely use scaling algorithms.

## 8. Conclusions

As shown in this paper, the use of minimum-redundancy residue code enables the construction of efficient scaling procedures based on the CRT due to optimizing the calculation of the rank of a number, a principal positional characteristic in RNS arithmetic.

At the beginning stage of the power-of-two scaling procedure, to calculate the rank of the initial number, we apply the approach for the rank calculation proposed by one of the authors in [33,34]. It is reduced to the summation of the small word-length residues $R_{1,k}\left(\chi_{1}\right)$, $R_{2,k}\left(\chi_{2}\right)$, …, $R_{k,k}\left(\chi_{k}\right)$, taking into account the number of occurred overflows during the modular addition operations modulo $m_{k}$, and fast calculation of two-valued rank correction $\delta_{k}\left(X\right)\in \left\{0,1\right\}$ (see (12) and (16)).

We propose a novel approach to power-of-two scaling based on Theorem 2. Using minimal residue code redundancy, we have optimized and sped up the rank calculation and parity determination of the numbers that result from division by two at each iteration of the bisection method. Each iteration of modular scaling by two is performed in only one modular clock cycle. Thus, owing to the proposed improvements, the power-of-two scaling procedure becomes simplest and faster than the currently known methods.

The computational complexity of the proposed scaling method by constant $S_{l}=2^{l}$ concerning required both modular addition operations and lookup tables is estimated as *k* and 2k+1, respectively, where *k* equals the number of primary non-redundant RNS moduli. The time complexity is $\left\lceil \log_{2}k\right\rceil +l$ modular clock cycles.

The use of minimally redundant RNS and a novel approach to calculating the rank of a number at each iteration of the bisection method enables a significant decrease in the power-of-two scaling computational complexity. Corresponding reduction factors concerning the required modular addition operations and lookup tables are given in Table 1, Table 2 and Table 3.

The proposed approach to power-of-two scaling coincides with the development vector of modern high-performance computing using RNS arithmetic. It enables the implementation of an extensive class of tasks in various areas of science and technology, first of all in cryptography and digital signal processing.

## Figures and Tables

**Table 1:** Dependence of the reduction factor $C_{LUT}\left(k\right)$ on the moduli number.

Reduction Factor	Moduli Number					
	k=5	k=10	k=15	k=20	k=25	k=30
$C_{LUT}$	1.73	3.05	4.32	5.59	6.84	8.10

**Table 2:** Dependence of the reduction factor $C_{MO}\left(k,l\right)$ on the moduli number *k* and scaling factor $S_{l}=2^{l}$.

Scaling Factor		Moduli Number					
$S_{l}$	l	k=5	k=10	k=15	k=20	k=25	k=30
8	3	12.00	21,00	29.00	36.75	44.40	52.00
16	4	16.00	28.00	38.67	49.00	59.20	69.33
32	5	20.00	35.00	48.33	61.25	74.00	86.67
64	6	24.00	42.00	58.00	73.50	88.80	104.0
128	7	28.00	49.00	67.67	85.75	103.60	121.33
256	8	32.00	56.00	77.33	98.00	118.40	138.67
512	9	36.00	63.00	87.00	110.25	133.20	156.00
1024	10	40.00	70.00	96.67	122.5	148.00	173.33

**Table 3:** Dependence of reduction factors $C_{MO}^{\prime }$ and $C_{LUT}^{\prime }$ on the moduli number *k*.

Reduction Factor	Moduli Number					
	k=5	k=10	k=15	k=20	k=25	k=30
$C_{MO}^{\prime }$	4.00	7.00	9.67	12.25	14.80	17.33
$C_{LUT}^{\prime }$	1.81	3.10	4.84	5.61	6.86	8.11

## Data Availability

Not applicable.
